# Cardiovascular Disease, Intensive Care, and Mortality in Coronavirus Disease 2019 Patients: A Meta-Analysis

**DOI:** 10.5152/TJAR.2021.21066

**Published:** 2022-04-01

**Authors:** Zouina Sarfraz, Azza Sarfraz, Muzna Sarfraz, Iqra Zia, Moosa Zulfiqar Ali, Radhika Garimella, Sameer Saleem Tebha, Hafiza Hussain, Zainab Nadeem, Gaurav Patel

**Affiliations:** 1 Department of Research & Publication, Fatima Jinnah Medical University, Lahore, Pakistan; 2 Department of Paediatrics & Child Health, Aga Khan University, Karachi, Pakistan; 3 CMH Medical College, Lahore, Pakistan; 4 Dr. NTR University of Health Sciences, India; 5 Department of Neurosurgery and Neurology, Jinnah Medical and Dental College, Karachi, Pakistan; 6 Aga Khan University Faculty of Medicine, Karachi, Pakistan; 7 Smt NHL Municipal Medical College, Gujarat, India

**Keywords:** Cardiovascular, COVID-19, intensive care, mortality, myocardial infraction

## Abstract

**Objective:**

Coronavirus disease 2019 is caused by severe acute respiratory syndrome coronavirus-2. The coronavirus disease 2019 pandemic has imparted an extraordinary burden on the intensive care services, which is likely to echo in pandemic and critical care management globally. We aim to meta-analyze mortality outcomes in cardiovascular disease patients and groups receiving corticosteroids therapy, intensive care admission status during coronavirus disease 2019 hospitalization and groups receiving corticosteroid therapy, and lastly, mortality outcomes in mechanically ventilated patients. Finally, we collate a coronavirus disease 2019 field algorithm for ST-elevation myocardial infarction critical care.

**Methods:**

PubMed databases were searched for relevant observational studies with MeSH terms including, “cardiovascular disease,” “COVID-19,” “intensive care,” “mortality,” and “mechanical ventilation.” A random-effect model was used to calculate the risk ratio, using RevMan V5.3.

**Results:**

A total of 67 622 patients were included with 10 076 participants in the cardiovascular disease group. Overall, the mean age of the participants in the studies was 60 ± 1.6 years and 52.1% were female. A higher death risk was found in cardiovascular disease patients during and after coronavirus disease 2019 infection (risk ratio = 2.43, 95% CI  = 1.74 to 3.41, *P* < .0001). Mechanical ventilation was likened to worsen mortality rates at any time during the hospital stay (risk ratio = 5.32, 95% CI = 3.89 to 7.29, *P* < .0001). Publication bias was not observed and high methodological qualities were included.

**Conclusions:**

Cardiovascular disease imparts a high burden on intensive care leading to high mortality among coronavirus disease 2019 patients. It is essential that myocardial infarctions in the acute care setting, and conditions such as hypertension and coronary artery diseases, are closely monitored while leading coronavirus disease 2019 hospitalization protocols.

## Main Points

- Cardiovascular disease imparts a high burden on intensive care and critical care services for coronavirus disease (COVID-19).
- In this meta-analysis, we analyze mortality outcomes in patients with cardiovascular disease, the clinical outcomes of corticosteroid therapy, and death risks in mechanically ventilated patients.
- Finally, we present a fresh COVID-19 field algorithm for ST-elevation myocardial infarction critical care.

## Introduction

Coronavirus disease 2019 (COVID-19) is caused by severe acute respiratory syndrome coronavirus-2 (SARS-CoV-2).^1^ First identified in Wuhan, China, COVID-19 is associated with a wide range of clinical manifestations including mild upper respiratory symptoms, acute respiratory distress syndrome, shock, and death.^1^ Diseased patients may suffer from severe complications requiring continuous monitoring in intensive care units (ICUs). The inception of the idea of critical care can be dated back to the Crimean War in 1850. In 1927, Water Dandy arranged for a special area to increase monitoring of postoperative neurosurgical patients.^2^ During the Second World War, Florence Nightingale, the Pioneer of Modern Nursing, dedicated a critical care provision area to soldiers who were seriously injured in the battle, which allowed for the close monitoring of patients.^3^ The true implementation of critical care as a medical specialty did not occur until the 1952 polio outbreak in Copenhagen, where patients who developed respiratory failure were isolated, provided optimum monitoring, and critical care by the utilization of tracheostomy and positive pressure ventilation.^3^ The term “Critical Care Medicine” was first introduced in the late 1950s at the University of Southern California.^2^ Critical care settings have drastically evolved from inception to date, particularly, in terms of radiologic and therapeutic advancements.^4^

Regardless of these developments, the ongoing COVID-19 pandemic has imparted an extraordinary burden on the critical care services, and the impact of this burden is likely to echo in pandemic and critical care management across the world. To combat the detrimental effects of the SARS-CoV-2 virus, various therapeutic agents have been approved for COVID-19. Notably, corticosteroids are under evaluation and in clinical use as a treatment for COVID-19 infections primarily due to their role in the reduced expression of the angiotensin-converting enzyme 2 (ACE2).^5^ In this meta-analysis, we aim to analyze mortality outcomes in cardiovascular disease (CVD) patients and groups receiving corticosteroids therapy, intensive care admission status during COVID-19 hospitalization and groups receiving corticosteroid therapy, and lastly, mortality outcomes in mechanically ventilated patients. Finally, we collate a COVID-19 field algorithm for ST-elevation myocardial infarction (STEMI) critical care.

## Methods

### Search Strategy and Selection

**Figure 1.**
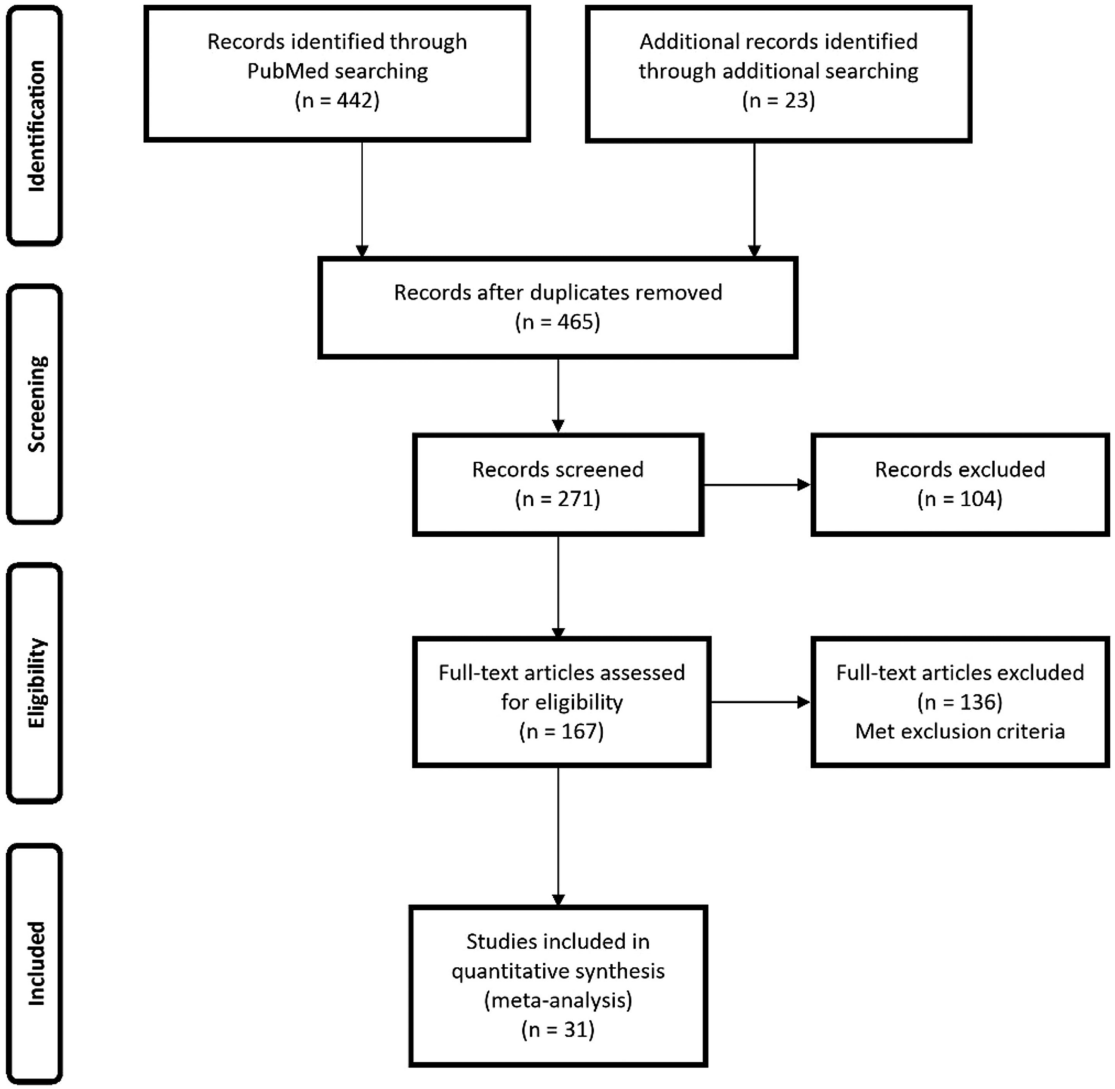
Preferred reporting items for systematic reviews and meta-analyses flowchart.

Preferred reporting items for systematic reviews and meta-analyses (PRISMA) guidelines were used to conduct the meta-analysis. PubMed databases were searched for relevant observational studies with MeSH terms including, “cardiovascular disease,” “COVID-19,” “intensive care,” “mortality,” and “mechanical ventilation.” The search was conducted from inception until January 1, 2021. Quantitative research articles were selected for the meta-analysis. All case reports, case series, letter to editors, reviews, systematic reviews, and meta-analyses were excluded. An umbrella method was employed where the reference lists of studies shortlisted for screening were assessed to ensure adequate inclusion of records. Journals including *NEJM*, *The Lancet Network*, and *The BMJ* were manually searched for relevant studies. Duplicates were removed using EndnoteX9 software by 2 independent reviewers (ZS and AS). During the first round of screening, the titles and abstracts were reviewed for significance. During the second round, full texts for shortlisted records were assessed. Any discrepancies throughout the screening and selection phase were resolved by active discussion by 2 reviewers (ZS and AS). In the third stage, discussions were resolved by all investigators, and the inclusion was sealed by the primary reviewer (ZS). Two reviewers tabulated data of the included studies in a shared spreadsheet (ZS and AS). Only high-quality methodological studies were included. The PRISMA flowchart is attached in Figure 1.

### Inclusion and Exclusion Criteria

The inclusion criteria included studies that enrolled patients > 18 years with diagnosed SARS-CoV-2 infection and CVD. The World Health Organization (WHO) definition for CVD was used throughout all studies included. Cardiovascular disease is the name for a group of disorders of blood vessels and heart and includes the following: (1) hypertension, (2) coronary heart disease, (3) cerebrovascular disease, (4) peripheral vascular disease, (5) heart failure, (6) rheumatic heart disease, (7) congenital heart disease, and (8) cardiomyopathies. The exclusion criteria were studies with no diagnosed COVID-19 patients and age < 18 years.

### Primary and Secondary Outcomes

The primary objective was to assess the mortality among COVID-19 patients with cardiovascular disease, mechanical ventilation, and receiving steroid treatment. The secondary outcome was to identify COVID-19 patients with CVD and steroid treatment among those admitted to ICUs.

### Statistical Analysis

A random-effect model using 95% CIs and significant *P* < .05 was used to calculate the risk ratio (RR), using RevMan V 5.3 (London, UK). The RRs of dichotomous measures between the included patient groups (CVD vs non-CVD) were presented as forest plots. Heterogeneity was identified using Higgin’s *I* ^2^. Funnel plots were generated to visually assess for publication bias.

## Results

A total of 67 622 patients were included with 10 076 participants in the CVD group. Overall, the mean age of the participants in the studies was 60 ± 1.6 years and 52.1% were female. The baseline demographic and comorbidities of the study population are shown in Table 1.

**Figure 2.**
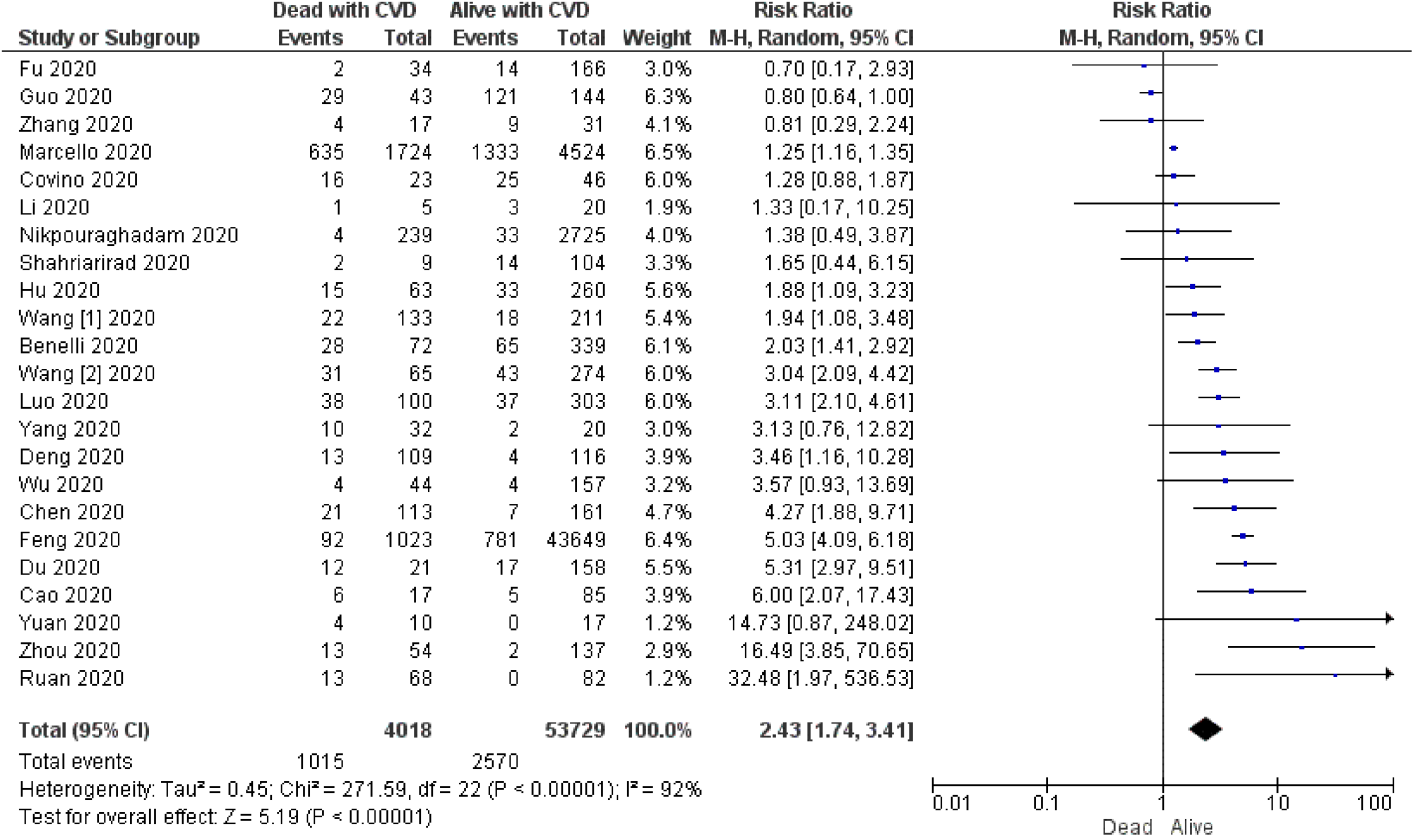
Forest plot of mortality outcomes in cardiovascular disease patients.

**Figure 3.**
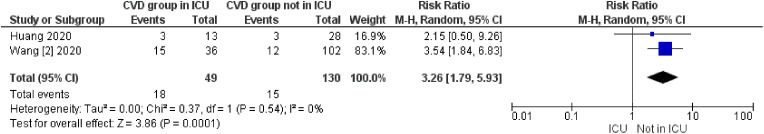
Forest plot of intensive care unit status and outcomes in cardiovascular disease patients.

**Figure 4.**
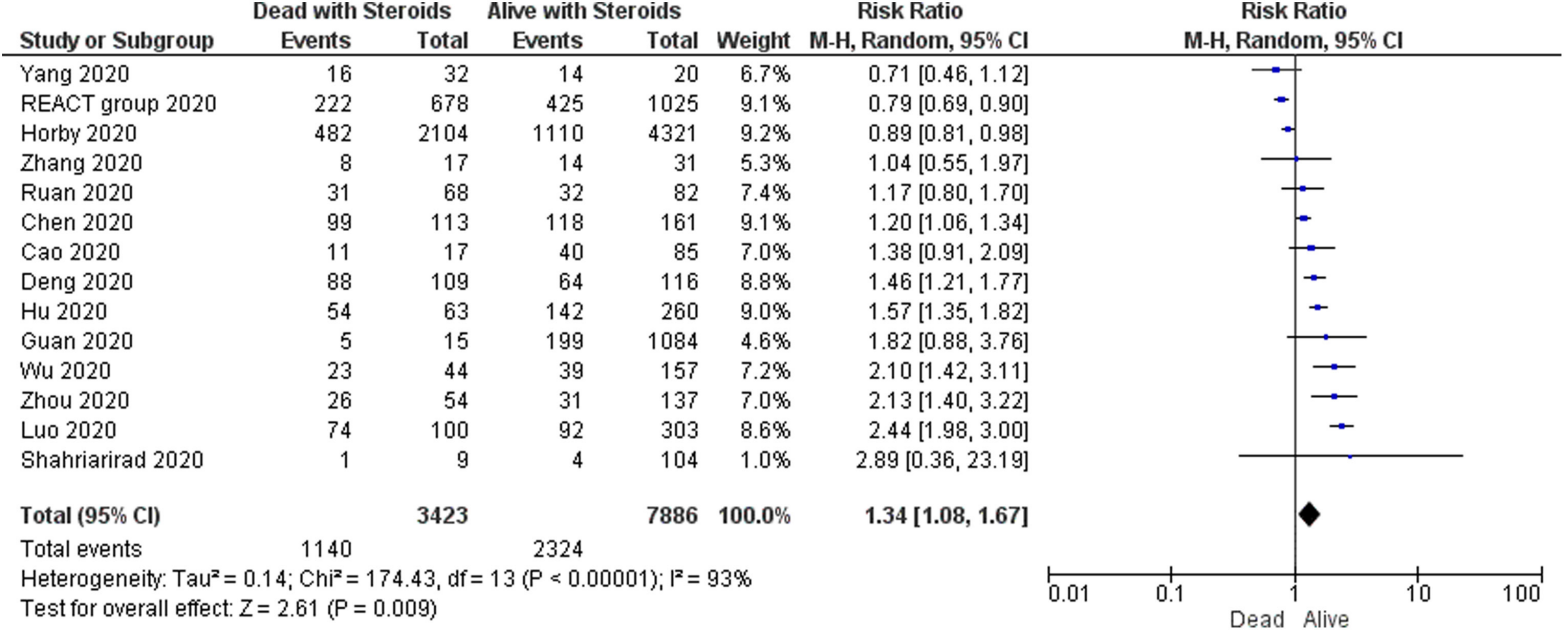
Forest plot of mortality outcomes in patient groups receiving corticosteroid therapy.

**Figure 5.**
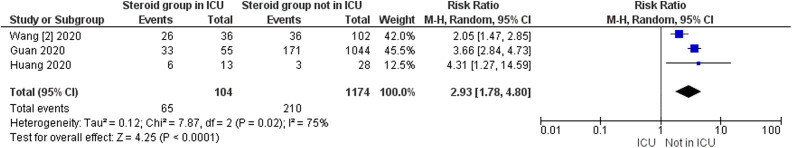
Forest plot of intensive care unit status and outcomes in patient groups receiving corticosteroid therapy.

**Figure 6.**
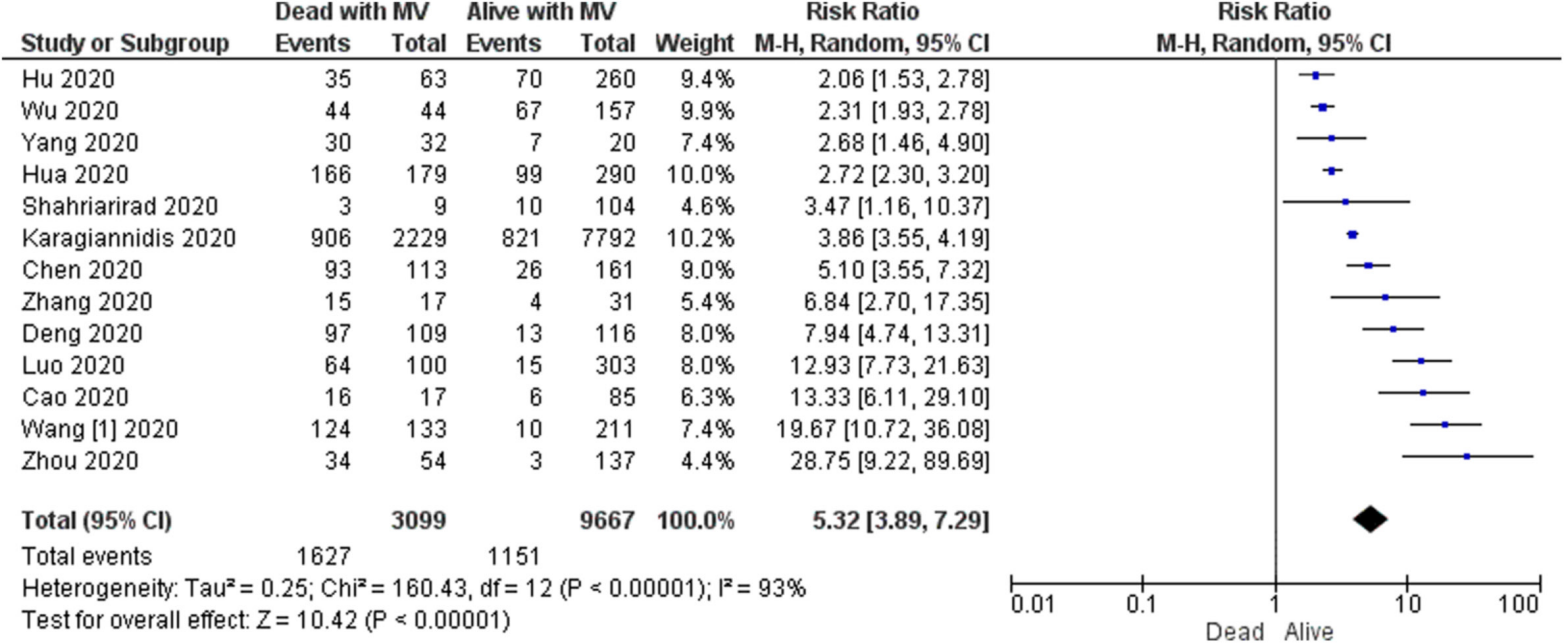
Forest plot of mortality outcomes in mechanically ventilated patients.

On noting mortality outcomes in patients with CVD, a higher risk of death was noted among the CVD group during and after COVID-19 infection (RR = 2.43, 95% CI  = 1.74 to 3.41, *P* < .0001) (Figure 2). The finding was statistically significant; however, there was high heterogeneity among the included studies (*I* ^2^ = 92%) (Figure 2). We found that patients with CVD had higher odds of being admitted to the ICU during hospitalization for COVID-19 infection (RR = 3.26, 95% CI = 1.79 to 5.93, *P * = .0001) (Figure 3). There was no heterogeneity among the included studies (*I* ^2^ = 0%) (Figure 3). The mortality outcomes in patients receiving steroids therapy were analyzed. Patients receiving steroids had an increased risk of mortality (RR = 1.34, 95% CI = 1.08 to 1.67, *P* = .009, *I* ^2^ = 93%) (Figure 4). Patients who received steroids were linked to ICU hospitalization (RR = 2.93, 95% CI  = 1.78 to 4.8, *P* < .0001, *I* ^2^ = 75%) (Figure 5). The mortality status of mechanically ventilated patients was attested. We find that patients were more likely to have high mortality if they were mechanically ventilated at any time during the hospital stay (RR = 5.32, 95% CI = 3.89 to 7.29, *P* < .0001, *I* ^2^ = 93%) (Figure 6). The included studies provide clear evidence that CAD patients have higher rates of being admitted to ICUs requiring more urgent intensive care and are associated with higher mortality. 

**Figure 7.**
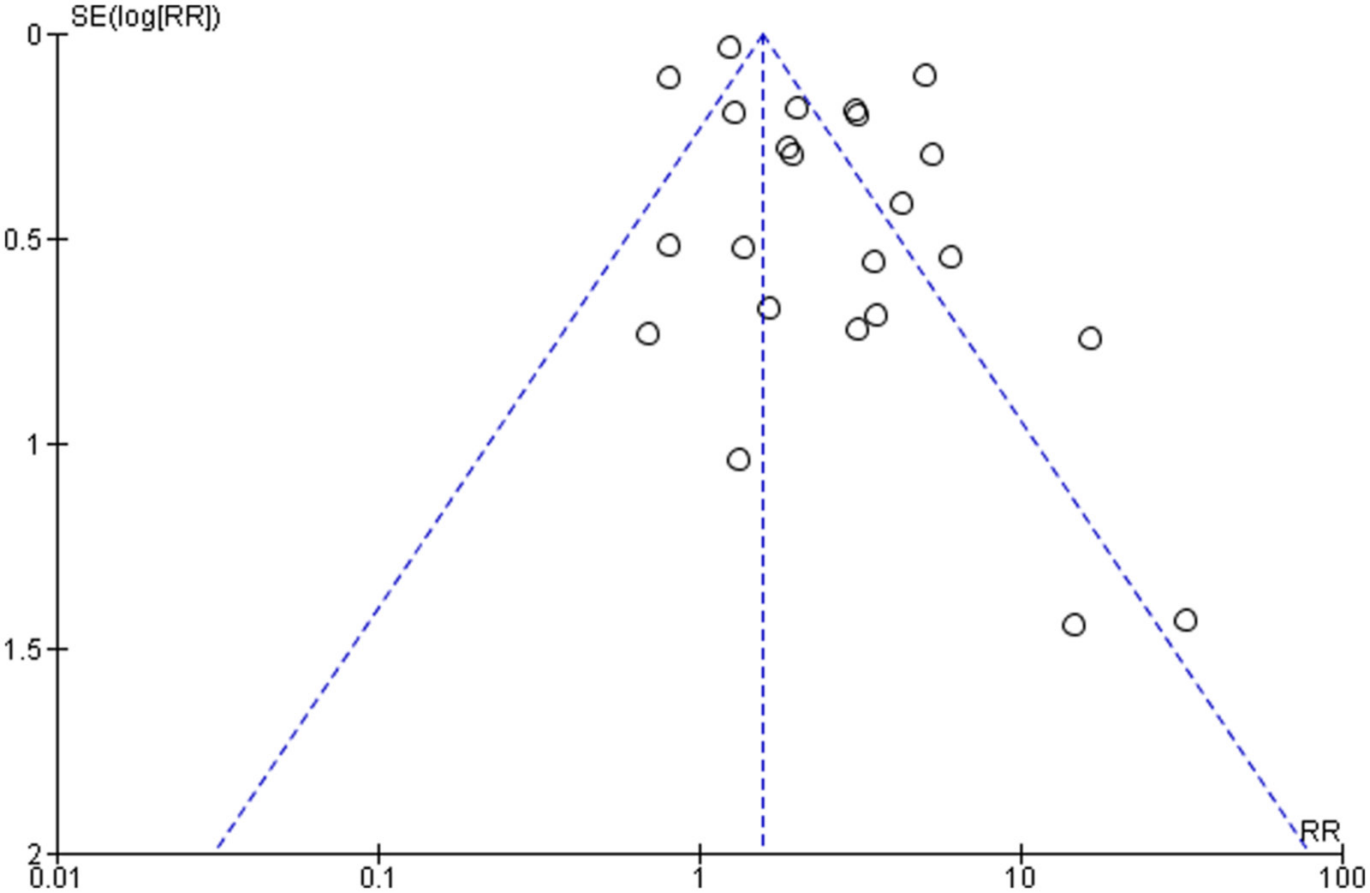
Funnel plot to assess publication bias of included studies in the meta-analysis.

The findings also identify a higher risk of intensive care and mortality among patients on cardiovascular drug therapy. Publication bias was not observed and high methodological qualities were identified in our analyzed studies (Figure 7).

## Discussion

The paper meta-analyzes intensive care and mortality outcomes among COVID-19 patients with CVD. Our pooled analysis of 67 622 patients finds that CVD patients have higher rates of being admitted to ICUs requiring more urgent intensive care, are associated with higher mortality. While benefits of corticosteroid therapy are typically observed during intensive care use, our meta-analysis finds that patients who received steroids had higher mortality trends (RR = 1.34, 95% CI = 1.08 to 1.67), possibly due to underlying CVD conditions. The WHO defines CVDs as a group of heart and blood vessel diseases including hypertension, coronary artery disease, heart failure, cardiomyopathies, and cardiac arrhythmias, which are also the number one cause of death globally.^6^ Cardiovascular mechanisms are linked to COVID-19 infections worldwide, which are likely to occur due to the interaction between the viral spike (S) glycoprotein and ACE-2.^7^ Severe acute respiratory syndrome coronavirus-2 utilizes the ACE-2 receptors expressed in the myocytes, coronary endothelial cells, and arterial smooth muscle to gain entry into host cells including type II alveolar epithelial cells and macrophages. Current literature well establishes that the SARS-CoV-2 infection utilizes the ACE2 receptors to gain entry into myocytes and coronary endothelial cells, ultimately leading to deleterious cardiovascular complications.^8^ As experiences of cardiovascular manifestations of COVID-19 increase, a higher number of patients will likely present with cardiac dysfunction or cytokine storm syndrome as arrhythmias, cardiomyopathies, or myocardial injuries.^9^

Our findings indicate that patients with CVD had a higher risk of death and grave mortality outcomes (RR = 2.43) in addition to being admitted to ICUs (RR = 3.26). Patients who received corticosteroids (RR = 2.93) or mechanical ventilation (RR = 5.32) were nearing end-of-life intensive care and were likely to have higher mortality. In the face of the growing COVID-19 pandemic, and the systemic disparities for minority populations contracting and dying from the viral infection, it is imperative to note the term, “post-intensive care syndrome.” The widespread risks for psychiatric, cognitive, and physical sequelae of critical illness among patients recovering from COVID-19 are discussed by Flash et al.^10^ During pandemics, health systems may temporarily collapse, ultimately requiring distinct admission criteria to ration the available services. While our results corroborate that patients with CVD and those under mechanical ventilation are more likely to die, the COVID-19 crises has raised the imperativeness of and the impetus to conduct goals-of-care discussion sessions with patients admitted to ICUs. The unprecedented escalation of patient loads in ICUs and emergency departments associated with the COVID-19 has accelerated end-of life discussions in select countries.^11^ While there is uncertainty about the potential outcomes of COVID-19 globally, the experiences so far have provided information about patients who require mechanical ventilation, that is, the association of old age (60 or above), high severity of illness determined by the WHO ordinal scale, respiratory distress, and a long duration of illness.^12^ In line with our findings, the outcomes of COVID-19 patients groups who require mechanical ventilation are poorer than those who do not (66.3% vs 19.4% in-hospital mortality).^13^

**Figure 8.**
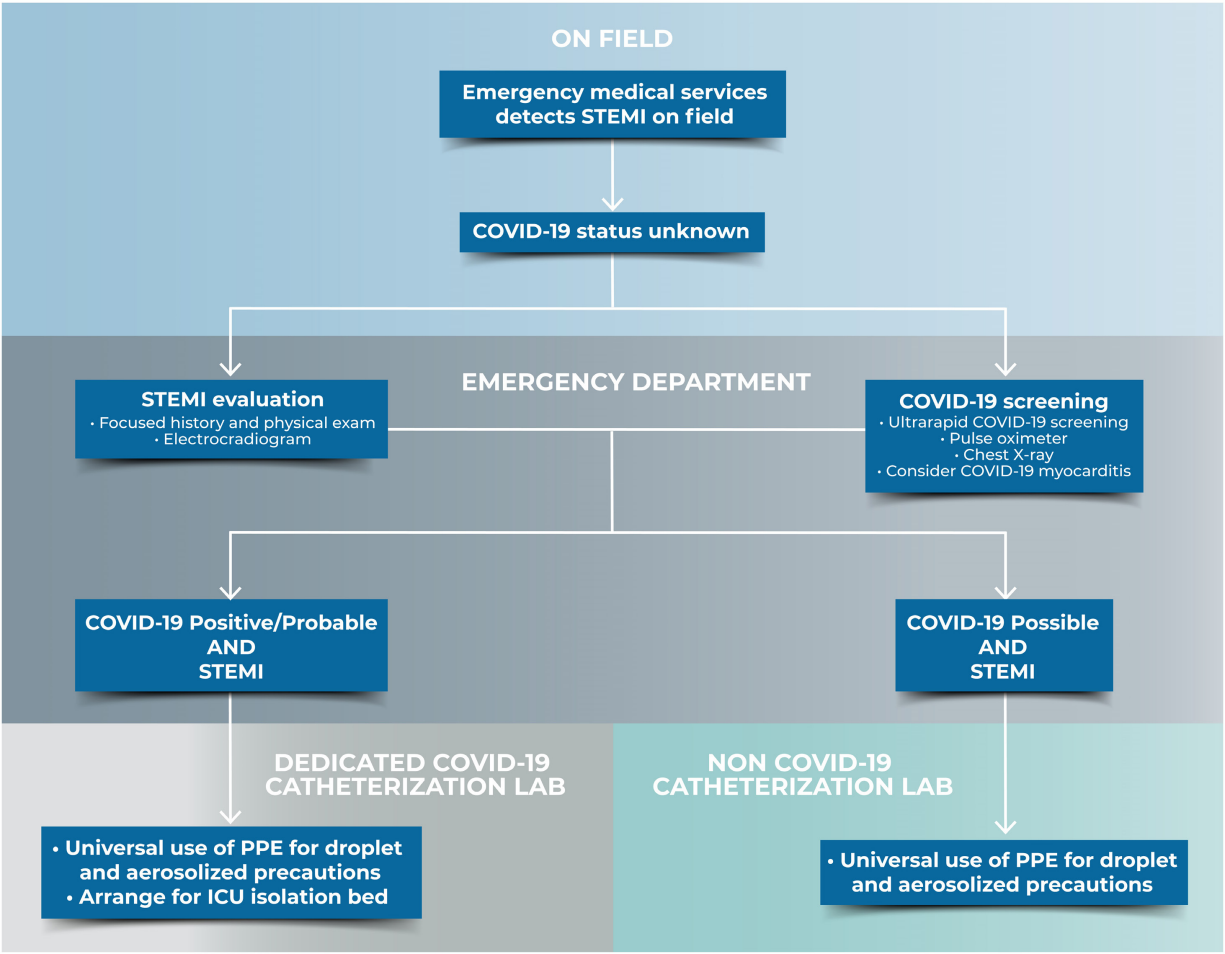
The protocol of care for ST-elevation myocardial infarction at non-percutaneous intervention, referral hospitals.^15^

**Figure 9.**
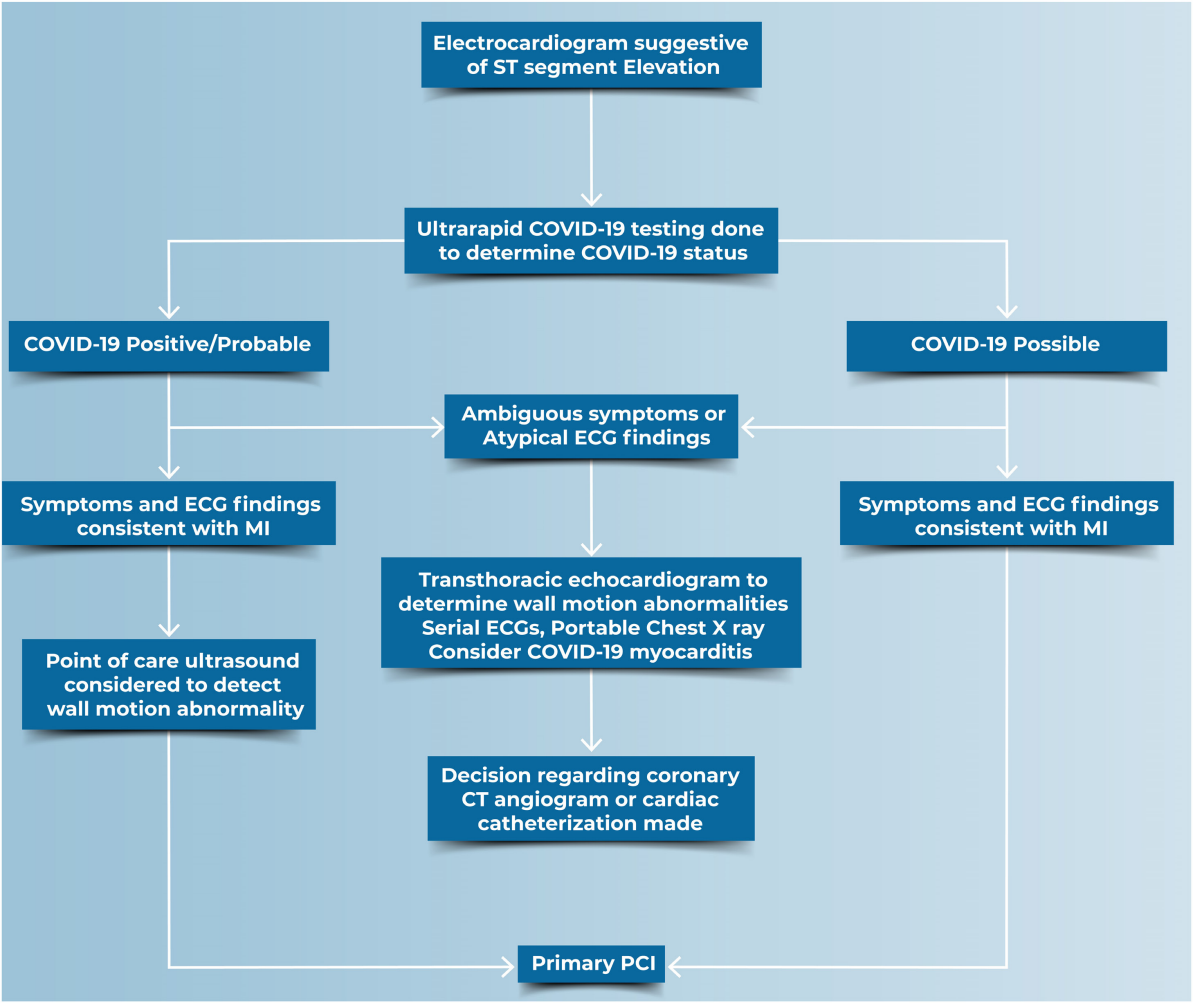
The approach to ST-elevation myocardial infarction: A coronavirus disease 2019 field algorithm.^15^

Despite many patients recovering from COVID-19, some reports highlight cardiovascular sequelae including heart damage due to inflammation.^14^ It is essential that these healthcare needs are met with a COVID-19 algorithm that may be employed during and post the pandemic. Devoted care teams have worked through the first wave of the COVID-19 pandemic to mitigate cardiac episodes ranging from cardiac fibrillation to basic arrhythmias to myocardial infarctions. While ICU capacities are exhausted or overrun, less attention has been paid to the after-effects of the virus, not only on cardiac patients but also for healthcare providers and organizations. The newer waves compounded with subacute and chronic cardiovascular sequelae of the prior waves of COVID-19 are likely to strain health systems due to the imminent cardiac after-effects. These cardiac episodes may be addressed via the Emergency Room (ER) and an established protocol of care for STEMI at non-percutaneous intervention, referral hospitals (Figure 8). The COVID-19 field algorithm for STEMI care describes the steps from an electrocardiogram suggestive of ST-segment elevation toward primary PCI. It has been depicted in Figure 9.

Our study has limitations. The lack of access to data such as the duration of CVD and time of diagnosis can particularly impact the ability to conduct risk stratification. Patients were diagnosed with multiple chronic diseases simultaneously, which could potentially impact the accuracy of the results. For select parameters, the heterogeneity among the studies for risk of mortality due to COVID-19 infection in patients with CVD was high, which could be a methodological shortcoming of the studies included in the meta-analysis. 

## Conclusion

In conclusion, our findings show that CVD impacts a high burden on intensive care admission and mortality among COVID-19 patients. It is essential that myocardial infarctions in the acute care setting and conditions such as hypertension and coronary artery diseases be closely monitored in intensive units during COVID-19 hospitalization protocols. The results of this study along with pertinent discussions may help improve the quality of cardiovascular and intensive care across healthcare systems worldwide, ultimately improving mortality outcomes of the COVID-19 pandemic.

### Declaration of Interest:

The authors have no conflict of interest to declare.

## Figures and Tables

**Table 1.** Characteristics of Included Patients

Total (n)	67 622
Female (n, %)	35 169 (52.1%)
Age, years (mean, SD)	60 (1.6)
Hypertension (n, %)	12 346 (20.8%)
Diabetes mellitus (n, %)	8490 (12.9%)
Former or current smoker (n, %)	385 (13.8%)
Chronic Kidney Disease (CKD)	3121 (13.5%)
